# DNA barcodes corroborating identification of mosquito species and multiplex real-time PCR differentiating *Culex pipiens* complex and *Culex torrentium* in Iran

**DOI:** 10.1371/journal.pone.0207308

**Published:** 2018-11-14

**Authors:** Nariman Shahhosseini, Mohammad Hassan Kayedi, Mohammad Mehdi Sedaghat, Trina Racine, Gary P. Kobinger, Seyed Hassan Moosa-Kazemi

**Affiliations:** 1 Département de Microbiologie-Infectiologie et d'Immunologie, Université Laval, Québec City, Québec, Canada; 2 Bernhard Nocht Institute for Tropical Medicine, WHO Collaborating Centre for Arbovirus and Hemorrhagic Fever Reference and Research, Hamburg, Germany; 3 Lorestan University of Medical Sciences, Department of Parasitology, Khorramabad, Iran; 4 Tehran University of Medical Sciences, Department of Medical Entomology and Vector Control, Tehran, Iran; 5 Department of Medical Microbiology, University of Manitoba, Winnipeg, Manitoba, Canada; 6 Department of Immunology, University of Manitoba, Winnipeg, Manitoba, Canada; 7 Department of Pathology and Laboratory Medicine, University of Pennsylvania School of Medicine, Philadelphia, PA, United States of America; Universidade de Sao Paulo, BRAZIL

## Abstract

Identifying mosquito species is a fundamental step in risk assessment and implementation of preventative strategies. Moreover, *Culex pipiens* is the most widespread mosquito vector in several regions of Iran and is the main vector for transmission of West Nile virus (WNV). Mosquitoes were collected at 14 sites in northern regions of Iran in 2015 and 2016. A subset of mosquito specimens was selected for identification confirmation using a DNA-barcoding technique. Construction of a phylogenetic tree showed clustering of mosquito sequences into three main genera: *Aedes*, *Anopheles* and *Culex* with individuals of a single species clustered closely together, regardless of where and when they were collected. *Cx*. *pipiens* complex and *Cx*. *torrentium* were identified and differentiated using multiplex real-time PCR targeting the gene locus for acetylcholinesterase 2 (ace2) to discriminate between *Cx*. *pipiens pipiens* and *Cx*. *torrentium*. The CQ11 microsatellite locus was used for discrimination between *Cpp*. biotypes. The predominant mosquito species in investigated regions were *Cx*. *pipiens pipiens* biotype *pipiens*, but we also detected *Culex pipiens pipiens* biotype *molestus* and hybrids of the two *pipiens* biotypes, as well as *Cx*. *torrentium*. The results of this study represent the first certain evidence of the presence of *Cx*. *pipiens pipiens* biotype *molestus* and hybrids between *pipiens* and *molestus* forms, and *Cx*. *torrentium* in Iran through a molecular identification approach. This report of a potentially important bridge vector for WNV might have key influence in the risk projections for WNV in Iran.

## Introduction

Many mosquito species are potential vectors for various pathogens and correct identification in all life stages is essential for effective mosquito monitoring and control [1]. By now, the checklist of Iranian mosquitoes (Diptera: Culicidae) includes 69 species representing seven genera [2–5], of which only a handful of species are known to be vectors for viral diseases [6]. The utilization of morphological characteristics is the most common method of mosquito species identification, but these characteristics can be difficult to interpret without specialized taxonomic expertise and discrimination among species is further complicated if identical characteristics are ruined [7]. For these reasons, a straightforward, reliable and easy-to-use identification method is always in high demand [8]. Although DNA barcoding has been demonstrated to be a well-founded technique for the identification of many taxa, including mosquitoes, some studies reported that two closely related mosquito species of the genus *Anopheles* and *Culex* could not be distinguished using the mitochondrial cytochrome c oxidase I (COI) barcode [9, 10].

Molecular assays to discriminate between *Cx*. *pipiens pipiens* (*Cpp*.) and *Cx*. *torrentium* or to distinguish between the *Cpp*. biotypes reported so far are based on gel electrophoretic analyses of particular DNA fragments amplified by PCR. These assays are time-consuming and prone to laboratory contamination, which are major disadvantages for the analysis of large sample sizes. To conquer those problems, we used a newly developed multiplex real-time PCR that allows simultaneous discrimination of the various morphologically indistinguishable *Culex* species and biotypes in a single PCR reaction. The assay targets the gene locus for ace2 to discriminate between *Cx*. *torrentium* and *Cpp*. and the CQ11 microsatellite locus for discrimination between *Cpp*. biotypes [11].

Recently, West Nile virus (WNV) has been reported in mosquitoes in northern Iran [12], and in humans in several parts of Iran [13, 14]. WNV belongs to the group of arthropod-borne viruses (arboviruses) and is transmitted through a mosquito bite. Accordingly, the spread of WNV is dependent on the presence of ornithophilic mosquito vectors in enzootic cycle, and knowledge of the local mosquito species and their distribution are important for assessing risks and for control of possible arbovirus outbreaks.

In some parts of Iran, the predominant mosquito species are the members of the *Cx*. *pipiens* complex, which serve foremost as vectors for various arboviruses, including WNV [15]. The *Cx*. *pipiens* complex includes several subspecies including *Cpp*. and *Cx*. *quinquefasciatus*, which are the most ubiquitous mosquitoes in temperate and tropical regions, respectively. *Cpp*. can be subdivided into two distinct biotypes: *pipiens* and *molestus*, which are morphologically indistinguishable and differ in physiology and behavior. Whereas, *Cpp*. biotype *pipiens* (Linnaeus, 1758) is ornithophilic (feeds on birds), is subjected to diapause (heterodynamic), is anautogeneous (only lays eggs after a blood-meal), and eurygamous (unable to mate in confined spaces), *Cpp*. biotype *molestus* (Forskal, 1775) is mammophilic (feeds on mammals), does not diapause (homodynamic), is autogeneous (lays first batch of eggs without taking a blood-meal) and stenogamous (mates in confined spaces). In addition, the *molestus* biotype is usually found underground in urban areas while the *pipiens* biotype always lives aboveground [16]. Identification of hybrids between *Cpp*. *pipiens* and *molestus* biotypes is important for WNV risk assessment because these hybrids indicate opportunistic host feeding preferences, which makes them a suitable vector for virus transmission [17].

The sibling species *Cx*. *torrentium* and *Cx*. *pipiens* are both compromised as vectors of enzootic bird-associated viruses [18]. Adult female *Cx*. *torrentium* have identical morphological characteristics as *Cpp*. biotype *pipiens* and *molestus*, which makes morphological discrimination of their females impossible [19].

Identification to the biotype level is considered of great epidemiological significance because *Cx*. *pipiens* complex has a wide distribution in Iran and the different biotypes exhibit different host feeding preferences, resulting in different vectoral capacity for WNV. The aim of this study was to complement morphological identification of a subset of specimens and assess the relative abundance of the *Cx*. *pipiens* biotypes in 14 different areas spread throughout three provinces of Iran.

## Materials and methods

### Mosquito collection and morphological identification

Mosquitoes were collected from 14 regions in private lands (the owner of the land gave permission to conduct the study on the site) with different habitat types (rice field, wildlife area, plant, swamp, riverside, wetland, shadow water) in three provinces (Guilan, Mazandaran and Golestan) in northern Iran from May to October 2015 and 2016 (S1 Table), as part of an arbovirus surveillance project. The field studies did not involve endangered or protected species. Various trap types were used, including BG-sentinel traps equipped with lure and CO_2_ sources, Gravid traps, and EVS traps equipped with an aspirator and dry ice as a source of CO_2_. Samples were shipped in a cool box to the Medical Entomology Laboratory at Tehran University of Medical Sciences for morphological identification of mosquitoes using discrimination keys of adult Culicidae mosquito species of Iran at the species level [20]. Samples from surveillance programs are often analyzed as pools of morphologically identical female mosquitoes, and as such, non-blood meal mosquitoes trapped in the same location over the same period were pooled up to 250 individuals, whilst blood-meal mosquitoes were labeled as individual samples. Individual blood-meal mosquitoes were investigated for blood-feeding pattern in another study. Later, morphologically identified female mosquito specimens were sent to Bernhard Nocht Institute for Tropical Medicine (Hamburg, Germany) for further molecular analysis.

### Confirming species identification through DNA-barcoding

The first mosquito-borne virus surveillance program in Iran in 2015–2016 recorded 25 morphologically distinct species in four genera [12]. A subset of morphologically identified blood-meal female mosquito species, comprising 55 individual mosquitoes, was selected for confirmation using DNA-barcoding. DNA-barcoding amplifies a 560 base pair (bp) fragment of the COI to identify species type of a wide range of mosquitoes. Each individual mosquito was homogenized and DNA extracted using the procedure previously described [21]. For PCR, amplification was conducted with primers targeting the mtDNA gene (560-bp fragment of COI). The primer pair used were: CI-J-1632 (5'-TGATCAAATTTATAAT-3') and CI-N-2191 (5'-GGTAAAATTAAAATATAAACTTC-3') [22]. Reactions were made in 25 μl volume containing 10 μl DNA template, 2× HotStar Taq plus Master Mix 4.20 μl, 25 mM MgCl2 1.6 μl, 10 μM of each primer in 0.6 μl (QIAGEN). The temperature profile consisted of an initial denaturation step at 95°C for 5 min, followed by 40 cycles of 94°C for 30 s, 40°C for 45 s, and 72°C for 1 min, and a final extension at 72°C for 10 min [23]. An aliquot of 5 μl of each PCR product was subjected to electrophoresis on a 2% agarose gel stained with Midori-green and photographed with a Gel Doc system. When bands with the expected size were visualized, the remaining PCR products were used for Sanger sequencing (LGC genomic, Berlin) and the sequences compared to already existing sequences from the publicly available database GenBank.

### DNA sequence analysis

In addition to sequences obtained in this study, a data set was extracted from GenBank (S2 Table). The DNA sequences were subjected to alignment using ClustalW. A neighbor joining (NJ) tree based on Tamura-Nei genetic distances was created to provide a graphic representation of the clustering pattern among different species [24, 25]. These analyses of the sequences were conducted using Geneious version 11.1.2 software. For comparison, the data set was also analyzed by Maximum Likelihood (ML) statistical method with Kimura 2-parameter model using MEGA version 7.0.26 software.

### Real-time PCR for differentiation of *Cx*. *pipiens* complex and *Cx*. *torrentium*

The species of female mosquitoes of the *Cx*. *pipiens* complex and *Cx*. *torrentium* were determined by a multiplex real-time PCR using previously designed primers for *Cx*. *pipiens*: 1725-F (5'-GCGGCCAAATATTGAGACTT-3') and 1726-R (5'- CGTCCTCAAACATCCAGACA-3') and probes: *Cx*. *pipiens* all (5'-Cy55- GGAACATGTTGAGCTTCGGK -BBQ-1–3'), *Cx*. *pipiens pipiens* biotype *pipiens* (5'-JOE-GCTTCGGTGAAGGTTTGTGT-BHQ1–3') and *Cx*. *pipiens pipiens* biotype *molestus* (5'- Rox-TGAACCCTCCAGTAAGGTATCAACTAC- BHQ2–3'). *Cx*. *torrentium* was detected using the primers *Cx*. *torrentium* F (5'-GACACAGGACGACAGAAA-3'), and R (5'- GCCTACGCAACTACTAAA-3') and the probe *Cx*. *torrentium* (5'-FAM- CGATGATGCCTGTGCTACCA-BHQ1-3'). Multiplex real-time PCR was carried out in a 20 μl reaction volume using HotStarTaq Master Mix Kit (Qiagen). Real-time PCR was performed with an initial denaturation step at 95°C (15 min), followed by 50 cycles of denaturation at 95°C (15 sec), annealing at 60°C (20 s), extension at 72°C (30 sec), and a final elongation at 40°C (30sec) [11].

## Results

### Mosquito barcoding

A total of 33,093 female mosquito specimens were collected including 32,618 non-blood meal female mosquitos in 387 pools and 475 individual female blood-meal mosquitoes (S3 Table), of which 55 individual female mosquitos were selected for identification confirmation by DNA-barcoding technique. Blasting sequence results in GenBank confirmed the identification of 5 *Ae*. *vexans*, 19 *An*. *pseudopictus/ hyrcanus*, 11 *An*. *sacharovi*, 10 *Cx*. *tritaeniorhyncus*, and 10 *Cx*. *pipiens*.

A NJ tree based on Tamura-Nei genetic distances showed clustering of mosquito sequences in three main genus *Aedes*, *Culex* and *Anopheles*, with individuals of a single species clustered closely together, regardless of where and when they were collected. Five species were characterized by a distinctive set of COI sequences that formed well-supported clusters in the NJ-tree (*Ae*. *unilineatus* used as outgroup). *Anopheles* clustered together, while *An*. *sacharovi* formed a separate group, and two sibling species, *An*. *hyrcanus* and *An*. *pseudopictus* formed a well-supported clade in the NJ tree, identified as monophyletic taxa.

**Fig 1:**
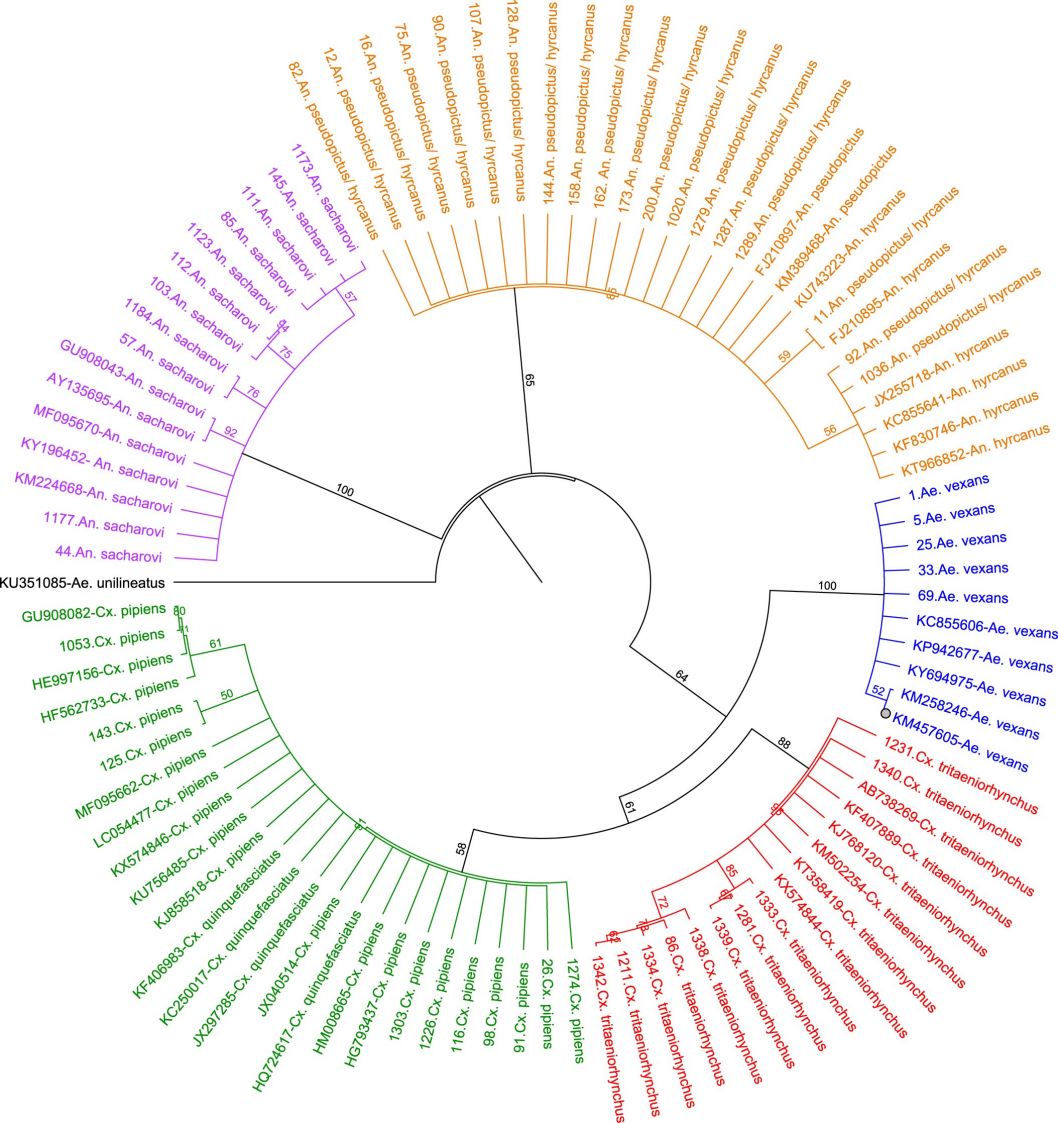
Phylogenetic tree based on COI sequences of 55 mosquitoes. An alignment of COI gene sequences (560 bp) was used to construct the NJ tree in Geneious 11.1.2 software. Numbers displayed on branches are the bootstrap support obtained through 1000 replications. Sequences obtained from GenBank are shown with accession numbers.

In the NJ tree, *Cx*. *pipiens* complex clustered with *Cx*. *tritaeniorhyncus* in *Culex* genus in two separate groups. *Cx*. *pipiens* and *Cx*. *quinquefasciatus* are two closely related species that can be confused using morphological identification. The samples of these two species were grouped together without any partition (Fig 1). In addition, comparison between phylogenetic trees constructed by NJ model (Geneious software) and ML model (MEGA software) showed similar topology (S1 Fig).

### Prevalence of *Cx*. *pipiens* complex and *Cx*. *torrentium*

A total of 21,170 (64%) specimens are identified as *Cx*. *pipiens* complex out of which 20,882 represent pooled samples (non-blood meal specimens) and 288 represent individual samples (blood-meal specimens). Overall, the predominant *Culex* species in Iran was found to be *cpp*. biotype *pipiens*, with an abundance of 15,205 (71.83%) pooled and individual specimens, whilst *cpp*. biotype *molestus* comprises 5 (0.02%) individual specimens, 4 collected with BG trap and 1 collected with EVS trap. The signal of *cpp*. biotype *pipiens* and *molestus* were found in 5,736 pooled specimens (27.09%), of which 10 (0.04%) individual specimens, all collected with BG-sentinel trap, can be considered as the hybrid *Cpp*. *pipiens*/*molestus* and the rest were a mixture of two biotypes in pooled samples. Also, multiplex real-time PCR results revealed 7 (0.03%) individual specimens were *Cx*. *quinquefasciatus*, 6 collected with BG-sentinel trap and 1 collected with EVS trap (Table 1).

**Table 1:** Number of collected *Cx*. *pipiens* complex and *Cx*. *torrentium* in Iran, 2015–2016.

Genus/ Species	Subspecies		Biotype	No. biotype/total *Culex* (%)	No. *Culex*/.total mosquitoes (%)
*Cx*. *pipiens* complex	*Cx*. *pipiens pipiens*		*pipiens*	15,205/ 21,170 (71.83%)	21,170/ 33,093 (64%)
			*molestus*	5/ 21,170 (0.02%)	
			*pipiens/molestus* hybrid	10/ 21,170 (0.04%)	
	*Cx*. *quinquefasciatus*		_	7/ 21,170 (0.03%)	
*Cx*. *torrentium*	_		_	2 mosquito pools	NA

Moreover, two mosquito pools containing 117 and 100 specimens showed a signal of both *Cx*. *pipiens and Cx*. *torrentium*, indicating the presence of *Cx*. *torrentium* among pooled specimens (Table 1).

### Dynamics of populations and species distributions

The greatest number of mosquitoes were collected in September (31.8%) followed by August (25%), July (18.9%) and June (17.7%), whilst the number of collected mosquitoes was less than 7% for May and October. Blood meal mosquitoes were mainly collected in July. No considerable regional differences were observed in *Culex* species abundance and species composition between trapping sites.

**Fig 2:**
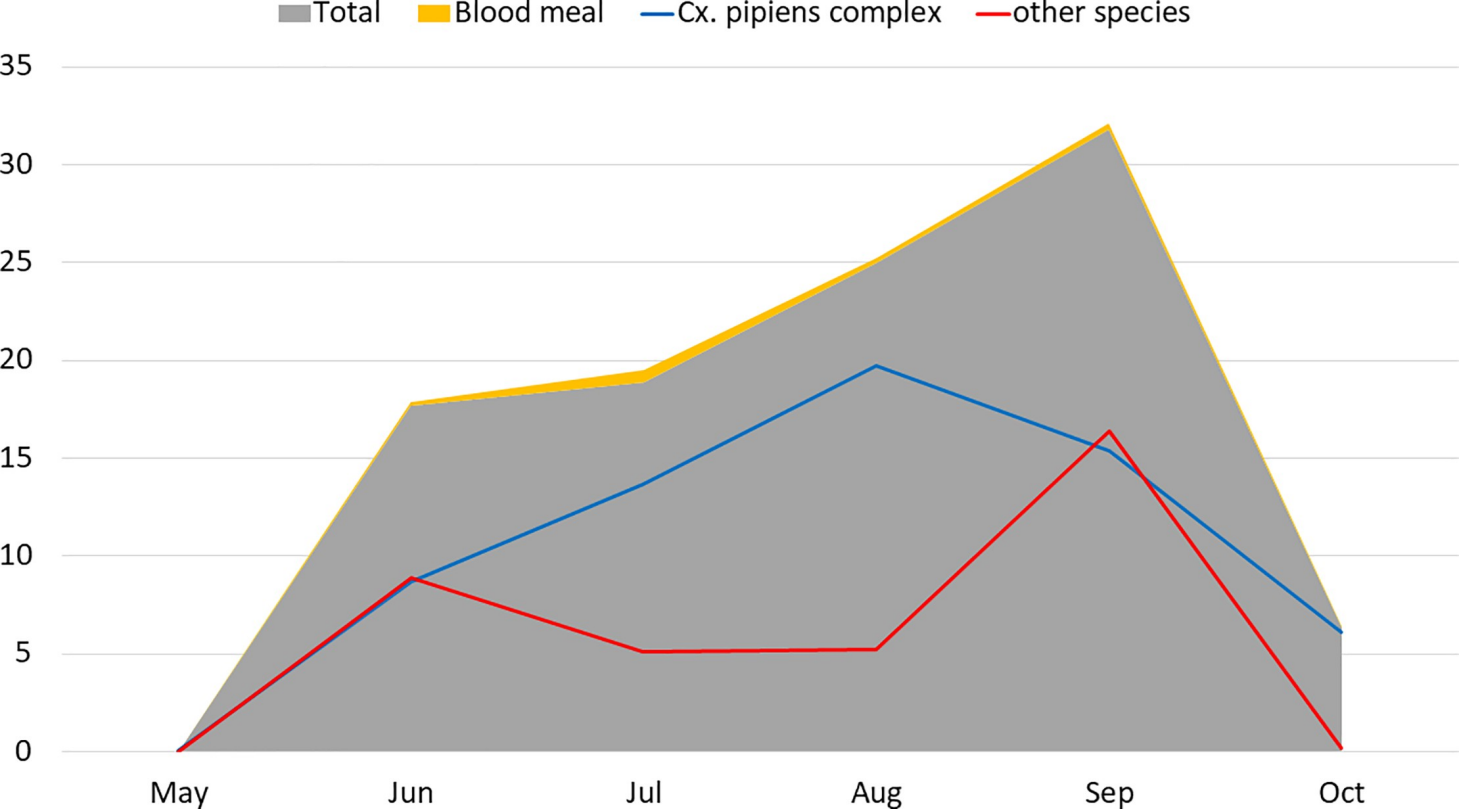
The percentage of total mosquitoes, blood-meals, *Cx*. *pipiens* complex and other mosquito species during trapping period in 2015 and 2016.

Regarding species dominating habitats between May and October, the prevalence of *Cx*. *pipiens* complex versus other mosquito species were almost equal in May, whereas *Cx*. *pipiens* complex became the dominant species in habitats for the next three months, peaking in August. In September, *Cx*. *pipiens* complex terminates its domination in habitats and its numbers fall, while the population of other mosquito species increase (Fig 2).

## Discussion

Several mosquito-borne viruses transmitted by culicine mosquitoes, such as Dengue virus and WNV, have been reported in Iran [26–29]. Since only a handful of mosquito species play an important role in disease transmission in Iran [30], precise mosquito identification and feeding behavior of mosquitoes are of note for monitoring, risk assessment and implementation of preventative strategies.

DNA barcoding is an efficient molecular technique for the identification of mosquitoes, despite the fact that identification of a few closely related species remains difficult [8].

The taxonomy of the species *An*. *hyrcanus* group still remains controversial, as *An*. *hyrcanus* and *An*. *pseudopictus* Grassi were originally treated as separate species [31]. Taking into consideration that *An*. *hyrcanus* has almost identical morphological features to *An*. *pseudopictus*, species identification via DNA barcoding would be an important addition to traditional morphology-based methods and a highly useful tool for species recognition [32]. Comparison of their COI sequences showed a high degree of genetic similarity and a phylogenetic tree could not be used to separate *An*. *hyrcanus* from *An*. *pseudopictus*, which is in accordance with a previous report [33]. Regarding the current genetic analyses, we agree with previous suggestions not to consider *An*. *hyrcanus* and *An*. *pseudopictus* as distinct species [34]. Moreover, member of the *Cx*. *pipiens* complex could not be distinguished based on their COI marker gene in phylogeny tree. Accordingly, the current sequence data based on COI marker demonstrated that a more precise molecular taxonomic technique is needed to differentiate mosquito species within *Cx*. *pipiens* complex, and *An*. *pseudopictus* and *An*. *hyrcanus* group.

Here we identify different morphologically indistinguishable *Culex* species and biotypes using a multiplex real-time PCR. The method was used to analyze 21,170 *Culex* specimens from a recent mosquito-borne virus surveillance program in Iran. The results indicate the existence of *Cx*. *pipiens* complex and *Cx*. *torrentium* in northern Iran, as well as the sympatric occurrence of *Cpp*. biotype *pipiens* and biotype *molestus*. Considering the low prevalence of biotype *molestus* within the investigated regions, it can be assumed that crossbreeding between the two *Cpp*. biotypes are a rare event.

Since pooled samples are not suitable for analysis of possible hybrids, a number of individual mosquito specimens were subjected to multiplex real-time PCR. As a result, for the first time ever, hybrids of *pipiens* and *molestus* biotypes were clearly identified in 10 individual specimens from Iran. Since the signal for *Cpp*. biotype *pipiens* and biotype *molestus* was detected in 27.09% (5,736) of pooled mosquito samples, it is likely that at least some of the pools that revealed a signal for *Cpp*. biotype *pipiens* and biotype *molestus* may contain biotype hybrids. To have a more realistic perspective for hybrid frequencies, more individual mosquito samples should be analyzed. The opportunistic blood-feeding preference of hybrids makes them high risk vectors to bridge WNV from avian to mammalian hosts [16]. Accordingly, the presence and abundance of hybrids in a region might increase the risk of WNV outbreaks in the human population [15].

While previous reports have demonstrated the presence of *Cx*. *quinquefasciatus* in south and central regions of Iran [35], the current data clearly indicates the presence of *Cx*. *quinquefasciatus* in the northern regions of Iran, a first for this species. There are some previous uncertain records of *Cx*. *torrentium* in Iran based on unreliable characteristics [2], but *Cx*. *torrentium* cannot be distinguished from members of the *Cx*. *pipiens* complex with certainty based on morphological characteristics alone [36]. Based on real-time PCR result, we confidently report the first evidence of *Cx*. *torrentium* in northern provinces of Iran.

Understanding the composition of mosquito populations is critical for evaluating their roles in disease transmission and developing more effective control strategies. According to the dynamics of species distributions, it can be assumed that WNV infections will occur more frequently in the investigated areas during July and August, when the *Cx*. *pipiens* population is at its peak, which also coincides with our ability to catch a high number of blood-engorged mosquitoes. This assumption is in accordance with previous findings of mosquito-derived WNV in August in a northern province of Iran [12].

It can be concluded that in northern provinces of Iran, *Cx*. *torrentium* evidence is solid, moreover, *Cx*. *pipiens* complex consists of *cpp*. biotype *pipiens*, *cpp*. biotype *molestus*, and a limited hybridization occur between them. Although *Cx*. *quinquefasciatus* was observed in the investigated areas, hybrids between *cpp*. biotype *pipiens* and *Cx*. *quinquefasciatus* was not detected. This report of a potentially important bridge vector for WNV might have pivotal impact in the risk projections for WNV in Iran.

## Supporting information

S1 Table Trapping sites in the Iran (2015–2016) with coordinate and land use information. (DOCX)

S2 Table GeneBank accession number of the specimens used in the sequence analysis. (DOCX)

S3 Table Species from the 475 blood-meal individual mosquitoes. (DOCX)

S1 Fig Phylogenetic tree was constructed by ML model based on COI sequences of 55 mosquitoes in MEGA 7.0.26 software. Numbers displayed on branches are the bootstrap support obtained through 1000 replications. Sequences obtained from GenBank are shown with accession numbers.
(TIFF)

## References

[1] Rozo-Lopez P, Mengual X. Mosquito species (Diptera, Culicidae) in three ecosystems from the Colombian Andes: identification through DNA barcoding and adult morphology. ZooKeys. 2015(513):39. 10.3897/zookeys.513.9561 PMC4524277 26257568

[2] Azari-Hamidian S. Checklist of Iranian mosquitoes (Diptera: Culicidae). Journal of Vector Ecology. 2007;32(2):235–42. 18260513 10.3376/1081-1710(2007)32[235:coimdc]2.0.co;2

[3] Djadid ND, Jazayeri H, Gholizadeh S, Rad SP, Zakeri S. First record of a new member of Anopheles Hyrcanus Group from Iran: molecular identification, diagnosis, phylogeny, status of kdr resistance and Plasmodium infection. Journal of medical entomology. 2009;46(5):1084–93. 19769039 10.1603/033.046.0515

[4] Azari-Hamidian S, Norouzi B, Noorallahi A. Orthopodomyia pulcripalpis (Diptera: Culicidae), a genus and species new to the Iranian mosquito fauna, with a review of bionomical information. Zootaxa. 2017;4299(1):141–5.

[5] Doosti S, Yaghoobi-Ershadi MR, Schaffner F, Moosa-Kazemi SH, Akbarzadeh K, Gooya MM, et al Mosquito surveillance and the first record of the invasive mosquito species Aedes (Stegomyia) albopictus (Skuse)(Diptera: Culicidae) in southern Iran. Iranian journal of public health. 2016;45(8):1064 27928533 PMC5139964

[6] Oshaghi M, Yaghobi-Ershadi M, Shemshad K, Pedram M, Amani H. The Anopheles superpictus complex: introduction of. Bull Soc Pathol Exot. 2008;101(5):429–34. 19192616

[7] Verna TN, Munstermann LE. Morphological variants of Aedes aegypti collected from the leeward island of Antigua. Journal of the American Mosquito Control Association. 2011;27(3):308–11. 10.2987/11-6157.1 22017096 PMC3357953

[8] Versteirt V, Nagy ZT, Roelants P, Denis L, Breman FC, Damiens D, et al Identification of Belgian mosquito species (Diptera: Culicidae) by DNA barcoding. Molecular Ecology Resources. 2015;15(2):449–57. 10.1111/1755-0998.12318 25143182

[9] Laurito M, de Oliveira TM, Almiron WR, Sallum MAM. COI barcode versus morphological identification of Culex (Culex)(Diptera: Culicidae) species: a case study using samples from Argentina and Brazil. Memórias do Instituto Oswaldo Cruz. 2013;108:110–22. 10.1590/0074-0276130457 PMC4109187 24473810

[10] Wang G, Li C, Guo X, Xing D, Dong Y, Wang Z, et al Identifying the main mosquito species in China based on DNA barcoding. PLoS One. 2012;7(10):e47051 10.1371/journal.pone.0047051 23071708 PMC3468562

[11] Rudolf M, Czajka C, Börstler J, Melaun C, Jöst H, von Thien H, et al First nationwide surveillance of Culex pipiens complex and Culex torrentium mosquitoes demonstrated the presence of Culex pipiens biotype pipiens/molestus hybrids in Germany. PloS one. 2013;8(9):e71832 10.1371/journal.pone.0071832 24039724 PMC3770594

[12] Shahhosseini N, Chinikar S, Moosa‐Kazemi SH, Sedaghat MM, Kayedi MH, Lühken R, et al West Nile Virus lineage‐2 in culex specimens from Iran. Tropical Medicine & International Health. 2017;22(10):1343–9. 28746985 10.1111/tmi.12935

[13] Meshkat Z, Chinikar S, Shakeri M, Manavifar L, Moradi M, Mirshahabi H, et al Prevalence of West Nile virus in Mashhad, Iran: A population–based study. Asian Pacific journal of tropical medicine. 2015;8(3):203–5. 10.1016/S1995-7645(14)60315-1 25902161

[14] Chinikar S, Shah-Hosseini N, Mostafavi E, Moradi M, Khakifirouz S, Jalali T, et al Seroprevalence of West Nile virus in Iran. Vector-Borne and Zoonotic Diseases. 2013;13(8):586–9. 10.1089/vbz.2012.1207 23697768

[15] Vogels CB, Fros JJ, Göertz GP, Pijlman GP, Koenraadt CJ. Vector competence of northern European Culex pipiens biotypes and hybrids for West Nile virus is differentially affected by temperature. Parasites & vectors. 2016;9(1):393. 27388451 10.1186/s13071-016-1677-0 PMC4937539

[16] Amraoui F, Tijane M, Sarih M, Failloux A-B. Molecular evidence of Culex pipiens form molestus and hybrids pipiens/molestus in Morocco, North Africa. Parasites & vectors. 2012;5(1):83. 22541050 10.1186/1756-3305-5-83 PMC3409039

[17] Reusken C, De Vries A, Buijs J, Braks M, Den Hartog W, Scholte EJ. First evidence for presence of Culex pipiens biotype molestus in the Netherlands, and of hybrid biotype pipiens and molestus in northern Europe. Journal of Vector Ecology. 2010;35(1):210–2. 10.1111/j.1948-7134.2010.00050.x 20618670

[18] LundstrÖM JO, Niklasson B, Francy DB. Swedish Culex torrentium and Cx. pipiens (Diptera: Culicidae) as experimental vectors of Ockelbo virus. Journal of medical entomology. 1990;27(4):561–3. 2167372 10.1093/jmedent/27.4.561

[19] Smith JL, Fonseca DM. Rapid assays for identification of members of the Culex (Culex) pipiens complex, their hybrids, and other sibling species (Diptera: Culicidae). The American journal of tropical medicine and hygiene. 2004;70(4):339–45. 15100444

[20] Azari-Hamidian S, Harbach RE. Keys to the adult females and fourth-instar larvae of the mosquitoes of Iran (Diptera: Culicidae). Zootaxa. 2009;2078(1):1–33.

[21] Shahhosseini N, Lühken R, Jöst H, Jansen S, Börstler J, Rieger T, et al Detection and characterization of a novel rhabdovirus in Aedes cantans mosquitoes and evidence for a mosquito-associated new genus in the family Rhabdoviridae. Infection, Genetics and Evolution. 2017;55:260–8. 10.1016/j.meegid.2017.09.026 28943405

[22] Kambhampati S, Smith P. PCR primers for the amplification of four insect mitochondrial gene fragments. Insect molecular biology. 1995;4(4):233–6. 8825760 10.1111/j.1365-2583.1995.tb00028.x

[23] Mousson L, Dauga C, Garrigues T, Schaffner F, Vazeille M, Failloux A-B. Phylogeography of Aedes (Stegomyia) aegypti (L.) and Aedes (Stegomyia) albopictus (Skuse)(Diptera: Culicidae) based on mitochondrial DNA variations. Genetics Research. 2005;86(1):1–11. 10.1017/S0016672305007627 16181519

[24] Kumar NP, Rajavel A, Natarajan R, Jambulingam P. DNA barcodes can distinguish species of Indian mosquitoes (Diptera: Culicidae). Journal of medical entomology. 2007;44(1):1–7. 17294914 10.1603/0022-2585(2007)44[1:dbcdso]2.0.co;2

[25] Cywinska A, Hunter F, Hebert PD. Identifying Canadian mosquito species through DNA barcodes. Medical and veterinary entomology. 2006;20(4):413–24. 10.1111/j.1365-2915.2006.00653.x 17199753

[26] Chinikar S, Ghiasi SM, Shah-Hosseini N, Mostafavi E, Moradi M, Khakifirouz S, et al Preliminary study of dengue virus infection in Iran. Travel medicine and infectious disease. 2013;11(3):166–9. 10.1016/j.tmaid.2012.10.001 23194952

[27] Shahhosseini N, Chinikar S, Nowotny N, Fooks AR, Schmidt-Chanasit J. Genetic analysis of imported dengue virus strains by Iranian travelers. Asian Pacific Journal of Tropical Disease. 2016;6(11):850–3.

[28] Shah-Hosseini N, Chinikar S, Ataei B, Fooks AR, Groschup MH. Phylogenetic analysis of West Nile virus genome, Iran. Emerging infectious diseases. 2014;20(8):1419 10.3201/eid2008.131321 25061976 PMC4111181

[29] Chinikar S, Shahhosseini N. Phylogenetic analysis on emerging Arboviruses in Iran. International Journal of Infectious Diseases. 2016;53:160.

[30] Chan A, Chiang L-P, Hapuarachchi HC, Tan C-H, Pang S-C, Lee R, et al DNA barcoding: complementing morphological identification of mosquito species in Singapore. Parasites & vectors. 2014;7(1):569. 25498759 10.1186/s13071-014-0569-4 PMC4282734

[31] Ramsdale C, Snow K. Distribution of the genus Anopheles in Europe. European Mosquito Bulletin. 2000(7):1–26.

[32] Ponçon N, Toty C, Kengne P, Alten B, Fontenille D. Molecular evidence for similarity between Anopheles hyrcanus (Diptera: Culicidae) and Anopheles pseudopictus (Diptera: Culicidae), sympatric potential vectors of malaria in France. Journal of medical entomology. 2008;45(3):576–80. 18533455 10.1603/0022-2585(2008)45[576:mefsba]2.0.co;2

[33] Fang Y, Shi W-Q, Zhang Y. Molecular phylogeny of Anopheles hyrcanus group members based on ITS2 rDNA. Parasites & vectors. 2017;10(1):417. 28882174 10.1186/s13071-017-2351-x PMC5590201

[34] Lebl K, Nischler EM, Walter M, Brugger K, Rubel F. First record of the disease vector Anopheles hyrcanus in Austria. Journal of the American Mosquito Control Association. 2013;29(1):59–60. 10.2987/12-6282.1 23687857

[35] Dehghan H, Sadraei J, Moosa-Kazemi S, Baniani NA, Nowruzi F. The molecular and morphological variations of Culex pipiens complex (Diptera: Culicidae) in Iran. Journal of vector borne diseases. 2013;50(2):111 23995312

[36] Danabalan R, Ponsonby DJ, Linton Y-M. A critical assessment of available molecular identification tools for determining the status of Culex pipiens sl in the United Kingdom. Journal of the American Mosquito Control Association. 2012;28(4s):68–74. 23401945 10.2987/8756-971X-28.0.68

