# Correction: DNA barcodes corroborating identification of mosquito species and multiplex real-time PCR differentiating *Culex pipiens* complex and *Culex torrentium* in Iran

**DOI:** 10.1371/journal.pone.0227018

**Published:** 2019-12-23

**Authors:** Nariman Shahhosseini, Mohammad Hassan Kayedi, Mohammad Mehdi Sedaghat, Trina Racine, Gary P. Kobinger, Seyed Hassan Moosa-Kazemi

The fifth author’s initials are indexed incorrectly in the article XML. The correct initials are Kobinger GP. The correct citation is: Shahhosseini N, Kayedi MH, Sedaghat MM, Racine T, Kobinger GP, Moosa-Kazemi SH (2018) DNA barcodes corroborating identification of mosquito species and multiplex real-time PCR differentiating *Culex pipiens* complex and *Culex torrentium* in Iran. PLoS ONE 13(11): e0207308. https://doi.org/10.1371/journal.pone.0207308
