# Supplementary material for: DNA barcodes corroborating identification of mosquito species and multiplex real-time PCR differentiating Culex pipiens complex and Culex torrentium in Iran
Source: PLoS One. 2018 Nov 14;13(11):e0207308. doi: 10.1371/journal.pone.0207308 (PMC6235353; doi:10.1371/journal.pone.0207308)
Supplement: S1 Table — (DOCX) [file pone.0207308.s001.docx]

| No | Province | Location | Latitude | Longitude | Landuse | Altitude |
| --- | --- | --- | --- | --- | --- | --- |
| 1 | Gilan | Anzali | 37° 27.759’ N | 049 27.893’ E | Rural/ Shadow water | -18 m |
| 2 | Gilan | Boujagh | 37° 25.186’ N | 049 55.942’ E | Natural/ Wildlife | -26 m |
| 3 | Gilan | Sepid-Rud | 37° 15.704’ N | 049 55.570’ E | Natural/ Riverside | -4 m |
| 4 | Gilan | Lahijan | 37° 12.444’ N | 050 01.360’ E | Urban/ plant | 9 m |
| 5 | Gilan | Doshman-Kord | 37° 21.906’ N | 050 05.433’ E | Rural/ Swamp | -22 m |
| 6 | Gilan | Dastak | 37° 22.850’ N | 050 06.701’ E | Rural/ Rice field | -14 m |
| 7 | Mazandaran | Tonekabon | 36° 50.179’ N | 050 50.198 E | Natural/ Forest | -13 m |
| 8 | Mazandaran | Chalus | 36° 41.190’ N | 051 23.842’ E | Natural/ Forest | -15 m |
| 9 | Mazandaran | Sisangan | 36° 35.031’ N | 051 47.803’ E | Natural | -15 m |
| 10 | Mazandaran | Molla-Kala | 36° 33.802’ N | 051 48.579’ E | Natural/ Mountain and Forest | 73 m |
| 11 | Mazandaran | Nur | 36° 34.134’ N | 051 57.673’ E | Natural/ Forest | -2 m |
| 12 | Mazandaran | Zareh | 36° 33.323’ N | 053 07.870’ E | Natural/ Forest Park | 60 m |
| 13 | Golestan | Miankaleh | 36° 50.970’ N | 053 24.781’ E | Natural/ Wildlife, Wetland | -16 m |
| 14 | Golestan | Bandar Gaz | 36° 46.946’ N | 053 56.668’ E | Rural/ Wetland | -16 m |
