# Supplementary material for: DNA barcodes corroborating identification of mosquito species and multiplex real-time PCR differentiating Culex pipiens complex and Culex torrentium in Iran
Source: PLoS One. 2018 Nov 14;13(11):e0207308. doi: 10.1371/journal.pone.0207308 (PMC6235353; doi:10.1371/journal.pone.0207308)
Supplement: S2 Table — (DOCX) [file pone.0207308.s002.docx]

| No. | GenBank accession No. | Species | Location |
| --- | --- | --- | --- |
| 1 | AB738269 | *Cx. tritaeniorhynchus* | Japan |
| 2 | AY135695 | *An. sacharovi* | Iran |
| 3 | FJ210895 | *An. hyrcanus* | Iran |
| 4 | FJ210897 | *An. pseudopictus* | Iran |
| 5 | GU908043 | *An. sacharovi* | Iran |
| 6 | GU908082 | *Cx. pipiens* | Canada |
| 7 | HE997156 | *Cx. pipiens* | Germany |
| 8 | HF562733 | *Cx. pipiens* | Germany |
| 9 | HG793437 | *Cx. pipiens* | Germany |
| 10 | HM008665 | *Cx. pipiens* | Germany |
| 11 | HQ724617 | *Cx. quinquefasciatus* | USA |
| 12 | JX040514 | *Cx. pipiens* | Sweden |
| 13 | JX255718 | *An. hyrcanus* | Tajikistan |
| 14 | JX297285 | *Cx. quinquefasciatus* | Mexico |
| 15 | KC250017 | *Cx. quinquefasciatus* | India |
| 16 | KC855606 | *Ae. vexans* | Russia |
| 17 | KC855641 | *An. hyrcanus* | Kazakhstan |
| 18 | KF406983 | *Cx. quinquefasciatus* | Pakistan |
| 19 | KF407889 | *Cx. tritaeniorhynchus* | Pakistan |
| 20 | KF830746 | *An. hyrcanus* | China |
| 21 | KJ768120 | *Cx. tritaeniorhynchus* | Pakistan |
| 22 | KJ858518 | *Cx. pipiens* | Turkey |
| 23 | KM224668 | *An. sacharovi* | Azerbaijan |
| 24 | KM258246 | *Ae. vexans* | Belgium |
| 25 | KM389468 | *An. pseudopictus* | Iran |
| 26 | KM457605 | *Ae. vexans* | Netherlands |
| 27 | KM502254 | *Cx. tritaeniorhynchus* | China |
| 28 | KP942677 | *Ae. vexans* | Sweden |
| 29 | KT358419 | *Cx. tritaeniorhynchus* | S. Korea |
| 30 | KT966852 | *An. hyrcanus* | China |
| 31 | KU743223 | *An. hyrcanus* | China |
| 32 | KU756485 | *Cx. pipiens* | Austria |
| 33 | KX574844 | *Cx. tritaeniorhynchus* | China |
| 34 | KX574846 | *Cx. pipiens* | China |
| 35 | KY196452 | *An. sacharovi* | Iran |
| 36 | KY694975 | *Ae. vexans* | Sweden |
| 37 | LC054477 | *Cx. pipiens* | Japan |
| 38 | MF095662 | *Cx. pipiens* | Turkey |
| 39 | MF095670 | *An. sacharovi* | Turkey |
| 40 | KU351085 | *Ae. unilineatus* | Iran |
