# Supplementary material for: DNA barcodes corroborating identification of mosquito species and multiplex real-time PCR differentiating Culex pipiens complex and Culex torrentium in Iran
Source: PLoS One. 2018 Nov 14;13(11):e0207308. doi: 10.1371/journal.pone.0207308 (PMC6235353; doi:10.1371/journal.pone.0207308)
Supplement: S3 Table — (DOCX) [file pone.0207308.s003.docx]

| Species | No. |
| --- | --- |
| *An. sacharovi* | 14 |
| *An. pseudopictus/ hyrcanus* | 59 |
| *Cx. tritaeniorhynchus* | 61 |
| *Cx. pipiens* | 335 |
| *Ae. vexans* | 6 |
| Total | 475 |
